# Altered Expression of TLR2 and TLR4 on Peripheral CD14+ Blood Monocytes in Children with Urinary Tract Infection

**DOI:** 10.1155/2016/6052891

**Published:** 2016-05-05

**Authors:** Panagiota Karananou, Alexandra Fleva, Despoina Tramma, Anastasia Alataki, Aikaterini Pavlitou-Tsiontsi, Maria Emporiadou-Peticopoulou, Efimia Papadopoulou-Alataki

**Affiliations:** ^1^4th Department of Pediatrics, Faculty of Medicine, Aristotle University of Thessaloniki and Papageorgiou General Hospital, Ring Road Nea Efkarpia, 56403 Thessaloniki, Greece; ^2^Immunology-Histocompatibily Department, “Papageorgiou” General Hospital, Ring Road Nea Efkarpia, 56403 Thessaloniki, Greece

## Abstract

Urinary tract infection (UTI) is the second most common bacterial infection, after otitis media, in infants and children. The mechanisms of disease susceptibility and the role of immunity in the pathogenesis of UTI in children have been evaluated. In recent years, Toll-Like Receptors (TLRs) have been recognized as specific components of the innate immune system constituting important mediators in host immune recognition. The aim of the present study was to determine ΤLR2 and TLR4 expression during the acute phase of UTI in infants and children by measuring the CD14/TLR2 and CD14/TLR4 expression on monocytes. We also attempted to compare the TLRs expression with the immunological status of the patients to healthy children. The study group consisted of 60 children (36 females and 24 males) and the control group included 60 age-matched pediatric subjects (27 females and 33 males). In our study, no antibody deficiency was found either in the children with UTI or in healthy subjects. There might be a connection between low IgA, IgG, and IgG subclasses serum levels and UTI as there was a statistically significant difference between patients and healthy children. A higher expression of CD14/TLR2 was revealed in patients (90,07%) compared to controls (85,48%) as well as CD14/TLR4 in patients (90,53%) compared to controls (87,25%) (statistically significant difference, *p* < 0,05). The results of this study could provide new understanding of UTIs' pathogenesis in children.

## 1. Introduction

Urinary tract infections (UTIs) are one of the most common infectious diseases in millions of people every year. They are triggered when infecting bacteria gradually establish in the bladder and multiply in the urine [1]. Recurrent UTI (rUTI) is a common problem in otherwise young healthy women: 27% to 44% of women experiencing an initial UTI develop rUTI [2].

In infants and children, UTI is the second most common bacterial infection, after otitis media [3]. The morbidity rate reaches up to 3% in prepubertal girls, 1% in prepubertal boys, and 8% in girls generally [4]. Urinary tract infections' clinical manifestations range from asymptomatic bacteriuria, acute cystitis, and other uncomplicated lower tract infections [5] to kidney infection, pyelonephritis, renal scar formation, hypertension, and secondarily the development of chronic renal failure. The management of children suffering from UTI has led to controversial guidelines over the last decades. Nowadays, new efforts have been made for less invasive treatment, imaging, and prophylactic antibiotics [6]. New horizons have been opened as far as the molecular basis. The mechanisms of disease susceptibility are also focused on the evaluation of the role of immunity in UTIs.

In recent years, the role of uroepithelial cells in response to UTI has been confirmed. It is a well grounded knowledge that uroepithelium is not just a physical barrier. It is also an active structure which triggers the inflammatory response and produces inflammatory cytokines against bacteria's and fimbrial structures [3]. Innate immunity is considered to be the front line of host defense against the multiplication of the pathogens [7]. It consists of locally secreted cytokines, proteins, and neutrophilic infiltration. The receptors which mediate host immune recognition and increase the expression of genes associated with inflammatory responses like TNF-a, IL-1, IL-6, and IL-12 were recently discovered and are known as Toll-Like Receptors (TLR) [8]. In bibliography, 13 TLRs have been reported in mammalian species [9]. They represent a family of transmembrane proteins and belong to the group of so-called Pattern Recognition Receptors (PRRs) [10]. TLRs are specific components of the innate immune system and constitute important mediators in host immune recognition [10]. UTI appears to be more relevant to TLR1, TLR2, and TLR4 [11]. They play a potential role in activating innate immunity with their early response against UTI and in protecting the mucosal barrier against attacks by bacteria. Uropathogenic* Escherichia coli* (*E. coli*) accounts for more than 85% of acute nonobstructive pyelonephritis and cystitis in uncompromised children [1].

So far, very few studies have indicated a relation between lower expression of TLR4 on monocytes in adult patients and recurrent UTIs [12]. In children, TLRs have been related only to asymptomatic bacteriuria by Ragnarsdóttir et al. [13] and to renal scarring by Bayram et al. [14]. The aim of the present study was to determine ΤLR2 and TLR4 expression during the acute phase of UTIs in infants and in children by measuring the CD14/TLR2 and CD14/TLR4 expression on monocytes. We also attempted to compare the TLRs expression to the immunological status of the patients with UTIs and healthy children.

## 2. Materials and Methods

Subjects were recruited from children who were admitted to the Pediatric Department or were investigated in the Outpatient Clinic of the 4th Department of Pediatrics, Faculty of Medicine, Aristotle University of Thessaloniki, Papageorgiou General Hospital. The study was retrospective and performed in children 6 months to 14 years old. The study was approved by the Ethical Committee of Aristotle University of Thessaloniki. All children were thoroughly physically examined and their medical history was evaluated in detail. They had complete blood count, measurement of urea and creatinine, Erythrocyte Sedimentation Rate (ESR), C-reactive protein (CRP), urinalysis, and urine microbiological analysis. A complete immunological profile was also assessed in every child including immunoglobulins, IgG IgA, IgM, IgG subclasses (IgG_1_, IgG_2_, IgG_3_, and IgG_4_), complement components C3 and C4, and peripheral blood immunophenotype, CD2, CD3, CD3+/CD4+, CD3+/CD8+, CD19, CD3−/16+56+, and CD3−/16+, CD3−/56+ cells and CD14/TLR2 and CD14/TLR4. A renal ultrasonography (USG) was finally performed on 45/60 patients.

### 2.1. Study Group (Group A)

It consisted of 60 children (36 females and 24 males), mean age of 3,3 ± 2,9 years, with at least one episode of acute urinary tract infection (AUTI) in their record, with or without underlying anatomic genitourinary anomalies. This group was divided into two subgroups: (A_1_) children with acute lower urinary tract infection and (A_2_) children with acute upper urinary tract infection.

Lower AUTI or cystitis was defined as the presence of a positive urine culture (colony count >10^5^ cfu/mL), with a urine white blood cell count of ≥ 25 cells per *μ*L (+1 with a dipstick), normal CRP (<0,8 mg/dL), ESR (<20 mm/h), and clinical manifestations such as the absence of fever, incontinence, dysuria, suprapubic pain, and malodorous urine. Upper AUTI or pyelonephritis was defined as a confirmed UTI, with a urine white blood cell count of ≥25 cells per *μ*L (+1 with a dipstick) and a positive urine culture (colony count > 10^5^ cfu/mL) plus the presence of ≥2 of the following criteria: fever (≥38°C), high CRP (>0,8 mg/dL) and/or ESR (>20 mm/h), and neutrophil levels above normal values for age [14, 15]. Clinical symptoms could also be abdominal or flank pain, malaise, nausea, vomiting, and occasionally diarrhea. Malformations of the urinary track were considered to be the following: vesicoureteral reflux, ectopic kidney, posterior urethra valves, duplicated pyelocaliceal system, and stenosis of the pyeloureteric junction.

### 2.2. Control Group (Group B)

The study also included 60 pediatric control subjects (27 females and 33 males), mean age of 7,01 ± 4,6 years, with no history of severe or chronic illness and no symptoms of acute infection. They had no recorded history of UTI and negative urine culture at the time of the collection of the samples. All control cases were recruited from the healthy children being followed up in the Outpatient Clinic.

### 2.3. Flow Cytometric Analysis

**Figure 1:**
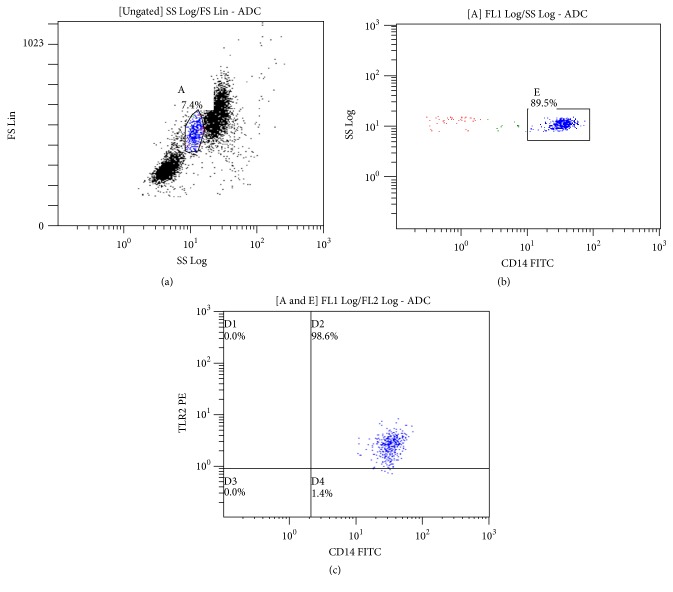
(a) Identified cell populations based on their physical characteristics. (b) Identification of CD14+ monocytes. (c) Double positive cells representing monocytes expressing TLR receptors.

Peripheral blood was collected in K_2_EDTA tubes and processed within 2 hours. TLRs, monocytes, and T-, B-, and natural killer (NK) cells were determined using two-color immunofluorescence staining with commercial monoclonal antibodies (eBioscience, Immunotech, and Coulter, resp.). Specifically, 100 *μ*L of whole blood was incubated with 20 *μ*L of appropriate monoclonal antibody and isotype control for 20 min in the dark at room temperature. The samples were then lysed by ImmunoPrep reagent system (Beckman Coulter Company) followed by flow cytometric analysis (FC 500, Beckman Coulter). Discrete cell populations were initially identified based on their physical characteristics on FSC versus SSC plot. Upon identification, gating on lymphocytes and the use of the appropriate antibodies determined the T-, B-, and NK cells. In order to further isolate and identify CD14 positive monocytes, after gating on monocytes, a second histogram was plotted as SSC versus CD14 fluorescence. Finally, a dual color parameter histogram was used (TLR versus CD14) to measure the double positive cells which represent the CD14 positive monocytes expressing TLR receptors (Figures 1(a), 1(b), and 1(c)).

### 2.4. Statistical Analysis

Data are expressed as mean ± SD or percentages for categorical variables. For continuous variables, nonparametric Mann-Whitney test was used, while Chi-squared test was employed for the comparison of two categorical variables. A *p* value less than 0.05 was considered statistically significant. SPSS version 20 has been utilized for all calculations.

## 3. Results

### 3.1. Demographic, Clinical Features and Laboratory Group Data

Demographic features of group A (patients) and group B (controls) are shown in Table 1. Sixty children with AUTI were evaluated. Eighteen patients were diagnosed with upper AUTI and 42/60 with lower AUTI. Forty-six patients had UTI only once while 14/60 patients suffered from UTI twice or more. Girls are affected by UTIs more frequently than boys. The majority of UTIs are lower UTIs in equal proportion between boys and girls. Girls suffered from upper UTIs more frequently than boys (Table 2).

Gram-negative bacteria were the causative pathogens in fifty-nine patients' (98,3%) urine cultures and only one (1,6%) was found with a Gram-positive urine culture (*Enterococcus faecalis*).* E. coli* was identified in the majority of the Gram-negative pathogens in forty-nine patients followed by* Pseudomonas aeruginosa* in six patients (10%) and* Proteus mirabilis* in four patients (7%). The renal USG revealed that 50% of the evaluated patients had pathological findings. Male children were found to have positive USG values twice those of the female children (Table 2). Vesicoureteral reflux and dilatation of pyelocaliceal system were the main findings and observed in 18/22 positive USG patients.

Laboratory data of group A (patients) and group B (controls) are shown in Table 3. The total count of leukocytes, as well as the percentage of neutrophils, was significantly higher in patients than in controls as expected. Both groups had normal mean serum creatinine levels matched for their age, although the serum creatinine level was higher in group B since group B consisted of older children. Finally, the CRP levels were statistically significantly higher in patients than in healthy children as expected (Table 3).

### 3.2. Expression of TLR2 and TL4 on Monocytes

The expression of CD14/TLR2 and CD14TL4 was measured in all 60 patients and 60 controls. The results of this counting revealed a significantly higher expression of CD14/TLR2 in patients than in controls. Similar results were observed in CD14/TLR4 (*p* < 0,05) (Table 4).

### 3.3. Immunophenotype of T- and B-Lymphocytes and NK Cells in Groups A and B

The CD3−/16+56+, CD3−/16+, and CD3/56+ count on natural killer cells did not show a statistically significant difference between patients and controls (*p* < 0,05). CD2 and CD3 counts on T-cells as well as T cytotoxic cells (CD3+/CD8+) count and CD19 count on B-lymphocytes have indicated an unimportant difference between patients and controls (*p* > 0,05). On the other hand, a slight statistical difference was confirmed, between patients and controls, as far as the count of the CD4 on T-cells and the CD4/CD8 ratio is concerned (Table 5).

### 3.4. Assessment of Immunoglobulins IgG, IgA, IgM, IgG Subclasses, and Complement Components (C3 and C4)

No primary immunodeficiency was detected in the subjects involved in this study. However, the concentrations of serum IgG, subclasses IgG1, IgG2, IgG3, and IgG4, and IgA in patients were statistically significantly lower than those in controls. No difference was noticed in the concentration of serum IgM. Finally, a higher measurement of C3 and C4 was found in patients compared to controls (Table 6).

## 4. Discussion

Although UTIs may seem to occur in a lower frequency in childhood than in adulthood, they are encountered among the most common bacterial infections in children. It is not yet fully understood why some of the children with a history of UTI progress to renal scarring, hypertension, renal impairment, and end-stage kidney disease while others do not. The identification of these patients is challenging and many studies and research works have been focused on this direction. During the 90s and early '00s, a great number of studies associated renal involvement with the presence and the grade of the vesicoureteral reflux or with other malformations of the urinary track, with the delay in treatment of acute pyelonephritis, and with the age of the first infection and the number of pyelonephritis incidents [15, 16]. During the last decade, early markers, genetic factors, and the role of immunity are being studied, indicating the complicated molecular interactions between bacterial virulence and host response [3].

The discovery of TLRs as part of the innate immunity and their role in host defense affirmed that innate immunity does not act in a generic way, as it was believed, but in more complex mechanisms. TLRs are considered to be the center of the immune response and are expressed in a great number of immune and nonimmune cells [17, 18]. Between them, TLR2 and TLR4 seem to be related to UTI. TLR2 identifies and interacts with a variety of microbial components such as lipopeptides, peptidoglycan, and lipoteichoic acid in Gram-positive bacteria and lipoproteins in mycoplasmas and mycobacteria. TLR4, on the other hand, is the lipopolysaccharide (LPS) signaling receptor [19–21].

CD14 is a 55 kDA glycoprotein mainly expressed by monocytes and macrophages and at a smaller extent by neutrophils and dendritic cells [19]. Although the participation of CD14 in activation of multiple signaling pathways is undoubtful, its role in host defense remains unclear [19]. Generally, CD14 on monocytes is considered to have a protective role against viral and bacterial infections. However, some studies have demonstrated both positive and negative effects of CD14 on infections, depending on the microorganism and the site of the infection [21, 22]. Many markers that are supposed to be involved in the activation of CD14, TLR4, and TLR2 on monocytes and macrophages during infections have also been studied. Shimizu et al. [21] investigated IL-8 induction by LPS in four bladder cancer cell lines and found that the presence of the membrane-anchored form of CD14 is a determinant of the inflammatory response of uroepithelial cells. Schilling et al. [23] studied the production of IL-6 and IL-8 by the interaction between type 1 piliated* E. coli* and bladder epithelial cells and revealed a high expression of CD14. In contrast, A498 renal epithelium cell line which was also studied and found to lack CD14 expression demonstrated poor IL-6 response to* E. coli*. Experiments on the contribution of CD14 to innate immune response have outlined the fact that the role of CD14 in host defense is not always protective [23–28].

There are many studies evaluating the relationship of CD14, TLR2, and TLR4 in various infections of adults. To our knowledge, there is no study investigating the relationship of CD14, TLR2, and TLR4 with acute UTIs in children. In our study, we made an effort to estimate the expression of TLR2 and TLR4 on monocytes (CD14) in children who suffered from UTI and compare them to healthy children. As stated previously in materials and methods, flow cytometric analysis identified CD14+ monocytes and discriminated them from NK cells and CD14+ granulocytes. Thus, we affirmed a higher expression of TLR2 and TLR4 on CD14 positive monocytes in children who have experienced one or more episodes of acute UTI than in children who had no UTI record in their medical background. This may suggest the signaling pathway activation which will promote the eradication of the bacteria from the urinary tract.

Immunological profile was assessed in patients and was compared to healthy children. In our study, the immunophenotyping that investigated the T- and B-lymphocytes and NK cells of the patients did not differ from that of healthy children (Table 5). However, the percentage of CD4 T-helper cells and the CD4/CD8 ratio were found to be slightly higher in patients than that in controls (*p* = 0,05) (statistically not significant) (Table 5). In order to further evaluate the immunity status of our subjects, we studied the serum levels of IgA, IgM, and IgG immunoglobulins and the subclasses of IgG (IgG1, IgG2, IgG3, and IgG4) in both study groups. None of our patients or controls had a primary or secondary immunodeficiency.

Few studies have been attempted to correlate immunodeficiencies with UTIs, in adults and not in children. According to Kutukculer et al. who studied 87 children (mean age of 46 ± 40,9 months) with antibody deficiencies, 3% of these children presented with UTI [29]. Kim et al. studied 55 adult patients with primary immunodeficiency disease. Infections of the genitourinary tract were among the least frequently observed infections and were mainly associated with IgG subclass deficiency [30]. In our study, no antibody deficiency was found either in the children with UTIs or in healthy subjects. However, there might be a connection between low IgA, IgG, and IgG subclasses serum levels and UTI as their levels were found statistically significantly lower in patients than in controls (Table 6). Finally, C3 and C4 serum levels were also measured in our study and were found within normal levels in both patients and healthy children. Many studies have been conducted to associate C3 and C4 component with UTI but their role remains debatable. In many reviews, it is stated that C3-deficient individuals are prone to infections caused by many bacteria among them and* E. coli* [31–34]. More recently, Syukri et al. [35] compared 34 young women with recurrent UTI and 34 healthy women and found that C3 levels were significantly lower compared with the levels of healthy women. These findings are controversial and do not come in accordance with the higher levels of C3 and C4 we found in children with UTI compared to the levels in children with no episode of UTI (statistically significant difference, Table 6). Overall, no primary immunodeficiency was detected in the subjects involved in this study.

There are certain noteworthy limitations in our work that we should consider. The study included a small cohort size of subjects that also lack prospective evaluation of monocyte/TLR2 and TLR4 expression in follow-up visits. Furthermore, we studied the expression of TLR2 and TLR4 exclusively on CD14+ monocytes. However, we are aware that CD14+ monocytes are an heterogeneous population and that there are distinct subsets that could alter the result of the expression of TLR. Finally, it should be mentioned that the significant variation of age we noted between the patients and the healthy group could also affect the tendency of variation in TLR expression. Since most of the children in the patients group were younger than those of the control group, a higher TLR expression in younger ages might need to be clarified. Further studies in large number of subjects should be performed to validate the effect of TLR2 and TLR4 on UTI. It would be also interesting if more studies and experiments could be contacted and perhaps measure, in children as well, the inflammatory markers that are produced from this signaling pathway (activation of CD14, TLR2, and TLR4).

In conclusion, our results indicate that children with acute UTI have a higher expression of TLR2 and TLR4 on monocytes. It seems that activation of the CD14/TLR2 and/or CD14/TLR4 pathway during the acute phase, which reflects the general activation of the immune system due to the underlying infection, may play an important role in the limitation of urinary tract infection. This finding might contribute to growing evidence of the role of TLRs expression in the pathogenesis of UTI in children.

## Figures and Tables

**Table 1:** Demographic features of group A (patients) and group B (controls).

	Group A	Group B
Gender		
Female	36 (60,0%)	27 (45,0%)
Male	24 (40,0%)	33 (55,0%)

Age (years) (mean ± SD)	3,3 ± 2,9	7,01 ± 4,6

**Table 2:** Demographic and clinical features of the patients.

Parameter	Number of patients		
	Total	Female	Male
Upper AUTI	18/60 (30%)	14/18 (77,7%)	4/18 (22,3%)

Lower AUTI	42/60 (70%)	22/42 (52,3%)	20/42 (47,7%)

Number of UTIs 1	46/60 (76,6%)	28/46 (60,8%)	18/46 (39,2%)
Number of UTIs ≥2	14/60 (23,3%)	8/14 (57,1%)	6/14 (42,9%)

Gram−/Gram+	59/1 (98,3%/1,6%)	36/0	23/1

USG (+)/(−)	22/23 (48,8%/51,1%)	9/16 (36%/64%)	13/7 (65%/35%)

UTI: urinary tract infection, AUTI: acute urinary tract infection, and USG: ultrasonography.

**Table 3:** Laboratory data of group A (patients) and group B (controls).

Parameters	Group A	Group B	*p*
	Mean ± SD		*t*-test
Leukocytes (×10^3^/u)	14462 (±6189)	8804 (±2749)	<0,001
Neutrophils (%)	54,3 (±18,20)	47,9 (±14,50)	0,0306
Serum creatinine (mg/dL)	0,474 (±0,079)	0,549 (±0,0941)	<0,001
CRP (mg/dL)	5,35 (±5,04)	0,52 (±0,47)	<0,001

**Table 4:** Percentages of TLR2 and TL4 on monocytes.

	Patients	Healthy children	*p* Mann-Whitney test
	Mean% ± SD		
CD14/TLR2	90,07 (±9,24)	85,48 (±10,94)	0.006
CD14/TLR4	90,53 (±8,87)	87,25 (±9,85)	0.044

**Table 5:** Percentages of T- and B-lymphocytes and natural killer cells in group A (patients) and group B (controls).

	Group A	Group B	*p* Mann-Whitney test
	Mean% ± SD		
CD3−/16+56+	9,87 (±4,86)	11,14 (±7,09)	ns
CD3−/16+	9,08 (±4,77)	10,09 (±6,83)	ns
CD3−/56+	7,43 (±4.49)	8.39 (±6,24)	ns
CD2	71,98 (±7,95)	73,10 (±6,20)	ns
CD3	66,10 (±7,91)	65,90 (±7,48)	ns
CD3+/CD4+	42,12 (±9,34)	39,24 (±6,78)	0.05
CD3+/CD8+	19,56 (±7,03)	21,77 (±6,11)	ns
CD19	21,74 (±8,22)	20,24 (±6,54)	ns
CD4/CD8	2,53 (±1,38)	1,98 (±0,77)	0.05

ns: not significant.

**Table 6:** Assessment of immunoglobulins IgG, IgA, and IgM, and IgG subclasses C3 and C4 in group A (patients) and group B (controls).

	Group A	Group B	*p* Mann-Whitney test
g/L	Mean (SD)		
IgG	6,83 (±3,10)	9,97 (±2,64)	<0.001
IgA	0,60 (±0,48)	1,12 (±0,80)	<0.001
IgM	0,93 (±0,58)	1,06 (±0,52)	ns
IgG1	5,01 (±2,38)	6,99 (±1,62)	<0.001
IgG2	1,30 (±0,74)	2,15 (±1,41)	<0.001
IgG3	0,31 (±0,17)	0,40 (±0,15)	0.004
IgG4	0,41 (±0,53)	0,83 (±0,93)	<0.001
C3	1,37 (±0,42)	1,15 (±0,24)	<0.001
C4	0,23 (±0,07)	0,19 (±0,05)	0.013

ns: not significant.
